# The role of sigmoid colon anatomic dimensions in the development of sigmoid volvulus, North-Western Ethiopia

**DOI:** 10.1371/journal.pone.0260708

**Published:** 2021-12-01

**Authors:** Agegnehu Berie Bayeh, Belta Asnakew Abegaz

**Affiliations:** 1 Department of General Surgery, College of Medicine and Health Sciences, Bahir Dar University, Bahir Dar, Amhara Regional State, Ethiopia; 2 Department of Biomedical Sciences, College of Medicine and Health Sciences, Bahir Dar University, Bahir Dar, Amhara Regional State, Ethiopia; Dipartimento di Scienze Mediche e Chirugiche (DIMEC), Orsola Hospital, ITALY

## Abstract

**Introduction:**

Sigmoid colon anatomic dimensions have been studied to have roles in the occurrence of sigmoid volvulus; however, these studies are few in number and failed to control the confounding effect of acute sigmoid obstruction on the anatomic dimensions. The main objective of this study was to assess the role of sigmoid colon anatomic dimensions in the development of sigmoid volvulus controlling the effect of acute sigmoid obstruction on the anatomic dimensions.

**Materials and methods:**

The study was carried out from Dec, 2019 to April, 2021 at Tibebe Ghion Specialized Hospital and Felege Hiwot Comprehensive Specialized Hospital, two referral hospitals in Bahir Dar city, North-Western Ethiopia to compare sigmoid anatomic dimensions among three independent groups of participants: patients with no history of sigmoid volvulus (I), those for whom elective surgery was done after non-surgical detorsion of sigmoid volvulus (II), and patients for whom emergency surgery was done for sigmoid volvulus (III). The anatomic dimensions were compared using fixed effects one-way ANOVA or Kruskal-Wallis H test at p-value ≤ .05 (two-sided) and Tukey method or Dunn-Bonferroni’s test was used for post-hoc comparisons.

**Results:**

A total of 66 consecutive eligible patients (22 for each of the three groups) were included and analyzed in the study. The means of anatomic dimensions (in cm) for groups (I, II, III) were: sigmoid colon length-SCL (35.91, 71.07, 80.86), meso-sigmoid height-MSH (17.11, 26.52, 28.86), meso-sigmoid maximal width-MSMW (9.70, 14.89,16.80), and meso-sigmoid root width-MSRW (8.34, 7.48, 8.11). SCL, MSH, MSMW, MSH/MSRW, and MSMW/MSRW were found to be statistically significantly different in patients with sigmoid volvulus. MSRW and MSH/MSMW were not different between the study groups.

**Conclusion:**

A long sigmoid colon with long and wide mesentery, but with a constant base is highly likely to predispose individuals to sigmoid volvulus.

## 1. Introduction

Sigmoid volvulus has been known to human since time immemorial. The ancient Egyptian medical text, Ebers papyrus and ancient Greek and Roman writings have detailed records of sigmoid volvulus [1]. It is an abnormal twist of the sigmoid colon on its own mesenteric axis [2]. It results in life threatening closed-loop obstruction [3].

Sigmoid volvulus as a cause of large bowel obstruction varies from 1 to 7% in the United States [4] to nearly 80% in the Andes [5]. Studies showed that Ethiopia has the highest incidence from Africa found to be a cause for more than 50% of intestinal obstructions with significant mortality and morbidity [6, 7].

Its etiology is multifactorial and controversial; but dietary, anatomic, and other factors have been hypothesized to predispose to this disease [8–15]. Worldwide, very limited number of studies on live patients (to the best knowledge of the authors only two) tried to compare anatomic dimensions of sigmoid colon between patients of sigmoid volvulus and controls. In these studies, longer sigmoid colon and dolicho-mesosigmoid (a mesentery longer than wide) were found in most patients with sigmoid volvulus than controls [12, 13], but patients with acute sigmoid volvulus (obstruction) were included in the studies as cases that the acute obstruction could contribute for the differences in the sigmoid colon anatomic dimensions between the study groups. Obstruction of bowel leads to an increase in its anatomic dimensions [16].

The main objective of this study was to assess the role of sigmoid anatomic dimensions in the development of sigmoid volvulus controlling the confounding effect of acute sigmoid obstruction on the anatomic dimensions, and it would be the first study of its kind to use elective patients with previous non-surgical deflation for sigmoid volvulus and for whom elective abdominal surgery under general anesthesia was done as one separate comparison group to rectify the limitation of the previous studies.

As the only and first study from a country in the ‘volvulus belt’ and with the highest incidence in Africa [6, 7], it provides evidences that refine the existing knowledge and understanding regarding the role that sigmoid anatomic dimensions play in the development of sigmoid volvulus and contributes to the body of knowledge in clinical anatomy, surgery, and gastroenterology. It can also be used as an ingredient for future multi-center and multi-ethnic researches.

## 2. Materials and methods

### 2.1 Study design, area, and period

The study was conducted at Tibebe Ghion Specialized Hospital (TGSH) and Felege Hiwot Comprehensive Specialized Hospital (FHCSH), two specialized referral public hospitals in Bahir Dar city, North-Western Ethiopia from Dec, 2019 to April, 2021. The city is located 587 km north-west of Addis Ababa, the capital city of Ethiopia. FHCSH was established in 1963. TGSH hospital serves as a teaching hospital for College of Medicine and Health Sciences (CMHS), Bahir Dar University (BDU) with several specialty and sub-specialty units and programs.

### 2.2 Ethics approval and consent to participate

A letter of approval/ethical clearance was obtained from Institutional Review Board (IRB) of CMHS, BDU. Written informed consent was obtained from each participant before the data collection and the study protocol was carried out in accordance with the declaration of Helsinki.

### 2.3 Sample size calculation

A total sample size of 66 (22 to each of the three groups) was calculated with a priori power analysis using g*power software version 3.1.9.2 for fixed effects one-way analysis of variance (ANOVA) to compare three means among three independent groups with a = .05, power (1- β)) = .80, and effect size f = 0 .4.

### 2.4 Inclusion and exclusion criteria

Group I included consenting adults (≥ 18 year-of-age) for whom abdominal surgery under general anesthesia was done for various reasons other than sigmoid volvulus on elective basis. The various indications for their surgery are presented in S2 Table.

Group II included consenting adults (≥ 18 year-of-age) with previous non-surgical detorsion (deflation) of clinically and radiologically confirmed sigmoid volvulus for whom abdominal surgery under general anesthesia was done on elective basis.

Group III included consenting adults (≥ 18 year-of-age) patients for whom emergency surgery was done for sigmoid volvulus following a failed attempt of conservative rectal deflation or a misdiagnosis for gangrenous volvulus or any other surgical emergency but intraoperatively found to have viable primary sigmoid volvulus.

Those with intra-operative hemodynamic instability, intra-peritoneal sepsis, gangrenous bowel, previous abdominal surgery, adhesions, and descending colon, sigmoid colon, rectal, and anal canal pathologies, and those in whom reaching sigmoid colon was not possible for technical reasons were excluded from the study.

### 2.5 Data quality assurance and data collectors

Senior residents in general surgery during the research period participated as data collectors. A one-day training was given to them on the purpose of the study and the procedures to be followed during data collection by the principal investigators who also supervised the whole process; and a pre-developed data recording format was used.

### 2.6 Data collection procedures and measurements

Before significant manipulation and resection of the bowel, anatomic dimensions of sigmoid colon such as SCL (from the descending colon-sigmoid junction to the recto-sigmoid junction along the antimesenteric border), MSH (from the tip to the base of the meso-sigmoid), MSMW (the largest route between the meso-sigmoid edges), MSRW (the distance between edges of the meso-sigmoid base) were measured in centimeters for consecutive patients who fulfilled the inclusion criteria using non-stretchable sterile measuring tape with proper splay of the mesentery and the bowel (S1 Fig). MSH/MSMW, MSH/MSRW, and MSMW/MSRW ratios were calculated. The measurements and age (in years), sex, height in centimeters (cms), weight in kilogram (kg), body mass index (BMI, in kg/m^2^), medical registration numbers and other demographic data of patients were recorded to a pre-developed data collection format.

### 2.7 Statistical analysis

The collected data were checked for completeness and entered in to SPSS version 21 for analysis. Boxplots, Shapiro-Wilk test, and Levene’s test were used to check outliers, normality of distributions, and homogeneity of variances of continuous variables, respectively. Continuous variables were compared using fixed effects one-way ANOVA, Welch’s ANOVA, or Kruskal-Wallis H test at p-value ≤ .05 (two-sided). Tukey method and Dunn-Bonferroni’s test were used for post-hoc multiple comparisons after significant one-way ANOVA and Kruskal-Wallis H test, respectively.

## 3. Results

### 3.1 Sociodemographic data of participants

A total of 66 patients (22 for group I, 22 for group II, and 22 for group III) were included and analyzed in the study. Most of the participants in each of the three comparison groups were males and from rural residence. Sixteen (72.73%) of group I, 21 (95.45%) of group II, and 17 (77.27%) of group3 patients were males. Eleven (50%) of group I, 20 (90.90%) of group II, and 19 (86.36%) of group3 patients came from rural areas. The three comparison groups were found not to be statistically significantly different in their ages and BMI as determined by one-way ANOVA (F (2,63) = 1.757, p = .181), and Welch’s ANOVA (F (2,40.341) =, p = .185), respectively (Table 1).

**Table 1:** Sociodemographic data of participants.

Group	Variables	Mean	95% CI for the mean		Median	SD	Minimum	Maximum
			Lower bound	Upper bound				
**Group I**	Age (in years)	47.27	40.53	54.02	50	15.21	20.00	70.00
	Weight (in kg)	61.68	58.84	64.53	61.25	6.42	50.00	74.00
	Height (in cm)	169.68	167.96	171.40	170	3.88	161.00	176.00
	BMI (in kg/m^2^)	21.19	19.84	22.55	21.39	3.06	15.36	26.23
**Group II**	Age (in years)	55.95	48.87	63.04	55	15.98	21.00	84.00
	Weight (in kg)	58.35	55.02	61.69	57.75	7.52	43.00	73.50
	Height (in cm)	170.5	167.21	173.79	170.5	7.43	150.00	185.00
	BMI (in kg/m^2^)	19.94	19.18	20.71	19.75	1.72	17.21	23.20
**Group III**	Age (in years)	52.23	45.56	58.89	55	15.03	22	75
	Weight (in kg)	57.18	55.03	59.33	56.5	4.85	51	70
	Height (in cm)	170.16	167.69	172.63	169.5	5.58	158	183
	BMI (in kg/m^2^)	19.79	18.96	20.62	19.84	1.88	16.10	22.41

### 3.2 Measurements of anatomic dimensions

The study showed that sigmoid colon length, meso-sigmoid height, and meso-sigmoid maximal width were averagely longer and higher in patients with sigmoid volvulus than those with no current and/or past clinical history of sigmoid volvulus. The meso-sigmoid root width (mesenteric base) was not statistically significantly different among the three groups. The average values of the three ratios of the sigmoid colon anatomic dimensions were also analyzed and summarized based on the comparison groups (Table 2).

**Table 2:** Sigmoid colon anatomic dimensions measurements.

Groups	N	Anatomic dimensions	Mean	Median	SD	95% CI for mean		Minimum	Maximum
						Lower bound	Upper bound		
**Group I**	22	SCL	35.91	36.25	10.52	31.24	40.57	18	60
		MSH	17.11	17.25	3.85	15.40	18.82	12	28
		MSMW	9.70	9.75	2.36	8.66	10.75	5	13
		MSRW	8.34	8.25	2.28	7.33	9.35	5	14
		MSH/MSMW	1.85	1.77	.53	1.62	2.09	1.08	3.20
		MSH/MSRW	2.17	2.20	.62	1.90	2.45	1.00	3.23
		MSMW/MSRW	1.24	1.23	.39	1.07	1.42	.39	1.8
**Group II**	22	SCL	71.07	70.00	8.44	67.32	74.81	55	87
		MSH	26.52	26.00	3.68	24.89	28.15	21	34
		MSMW	14.89	15.50	3.32	13.41	16.36	9	20
		MSRW	7.48	7.75	1.58	6.77	8.18	5	11
		MSH/MSMW	1.87	1.71	.55	1.63	2.12	1.25	3.40
		MSH/MSRW	3.70	3.57	.97	3.27	4.13	2.60	6.60
		MSMW/MSRW	2.08	2.00	.73	1.76	2.41	1.20	4.00
**Group III**	22	SCL	80.86	80.50	10.07	76.40	85.33	60	98
		MSH	28.86	29.00	3.68	27.23	30.50	23	36
		MSMW	16.80	17.00	3.55	15.22	18.37	11	25
		MSRW	8.11	7.00	2.05	7.20	9.02	6	13
		MSH/MSMW	1.78	1.65	.41	1.60	1.97	1.20	2.58
		MSH/MSRW	3.72	3.61	.82	3.35	4.08	2.31	5.54
		MSMW/MSRW	2.15	2.00	.55	1.90	2.39	1.40	3.38

### 3.3 Assumptions tests results

Assessment using boxplots showed that there were no outliers for SCL, MSH, MSW, and MSRW in all the three groups, but outliers were detected for MSH/MSW, MSH/MSRW, and MSW/MSRW in group II.

Normality assessment using Shapiro-Wilk test showed that distributions of SCL, MSH, and MSMW were normal in all groups, but those of MSRW in group III, MSH/MSMW in groups I and II, MSH/MSRW in group II and MSMW/MSRW in group II were found to be not normally distributed. Levene’s test for homogeneity of variances showed that the assumption was met for SCL, MSH, MSMW, MSRW, MSH/MSMW, MSH/MSRW, and MSMW/MSRW (Table 3).

**Table 3:** Assumptions tests for equality of variances and normality of distributions.

Variables	N	Levene’s F test for equality of variances		Shapiro-Wilk test for normality of distributions		
		F	p-value	p-value		
				Group I	Group II	Group III
SCL	22	.414	.663	.932	.798	.497
MSH	22	.029	.972	.083	.188	.524
MSW	22	1.824	.170	.175	.376	.856
MSRW	22	1.642	.202	.264	.514	.010
MSH/MSW	22	.190	.828	.026	.001	.152
MSH/MSRW	22	1.086	.344	.678	.008	.774
MSW/MSRW	22	1.557	.219	.246	.001	.083

Test of homogeneity of variance using Levene’s test based on median and with adjusted df showed that the groups had same distributions for MSRW, MSH/MSMW, MSH/MSRW, and MSMW/MSRW with p-values: .360, .859, .383, and .336, respectively.

One-way ANOVA for comparison of means of SCL, MSH, and MSMW; and Kruskal-Wallis H test for comparison of medians of MSRW, MSH/MSMW, MSH/MSRW, and MSMW/MSRW were used.

There was a statistically significant difference in SCL among the groups as determined by one-way ANOVA (F (2,63) = 130.131, p < .001, partial eta^2^ = .805). A Tukey post-hoc test revealed that group II patients had a statistically significantly longer SCL than group I patients, and patients in group III had a SCL statistically significantly longer than those in groups I and II.

One-way ANOVA showed that MSH and MSMW were statistically significantly different among the groups (F (2,63) = 60.864, p < .001, partial eta^2^ = .659, and F (2,63) = 30.433, p < .001, partial eta^2^ = .491), respectively. A follow up test using Tukey method showed that both were statistically significantly longer in group II than group I and in group III than group I. The differences between groups II and III did not amount to statistical significance.

As determined by Kruskal-Wallis H test, MSRW and MSH/MSMW were not statistically different among the groups, χ^2^ (2) = 1.575, p = .455, epsilon^2^ = .024, and χ^2^ (2) = .178, p = .915, epsilon^2^ = .003, respectively.

A Kruskal-Wallis H test showed that there were statistically significant differences in MSH/MSRW and MSMW/MSRW among the three groups, χ^2^ (2) = 34.228, p < .001, epsilon^2^ = .53 and χ^2^ (2) = 29.667, p < .001, epsilon^2^ = .46, respectively with a mean rank of MSH/MSRW of 14 for group I, 42.09 for group II, and 44.41 for group III; and a mean rank of MSMW/MSRW of 15.41 for group I, 40.98 for group II, and 44.11 for group III. The Dunn’s post-hoc test with Bonferroni correction revealed that the difference was statistically significant between groups I and II, and groups I and III, but not between Groups II and III for both ratios (Table 4).

**Table 4:** Pair-wise multiple comparisons.

Anatomic dimensions	Tukey’s HSD pairwise tests	Mean difference	p-value	95% CI for mean difference		Cohen’s d
				Lower bound	Upper bound	
**SCL**	II-I	35.16	< .001	28.12	42.19	3.69
	III-I	44.95	< .001	37.92	51.99	4.37
	III-II	9.80	.004	2.76	16.83	1.05
**MSH**	II-I	9.41	< .001	6.70	12.12	2.50
	III-I	11.75	< .001	9.04	14.46	3.12
	III-II	2.34	.103	-.3652	5.047	0.64
**MSMW**	II-I	5.18	< .001	2.9241	7.4396	1.80
	III-I	7.0909	< .001	4.8331	9.3487	2.36
	III-II	1.9091	.113	-.3487	4.1669	0.56
**Dunn-Bonferroni’s pairwise test**						
**Comparison groups**		II-I		III-I		III-II
**MSH/MSRW**	Adjusted significance	< .001		< .001		1.000
**MSMW/MSRW**	Adjusted significance	< .001		< .001		1.000

## 4. Discussion

As country in the volvulus belt, which includes parts of Africa, Middle East, Asia, Eastern Europe, and South America, Ethiopia has the highest report of sigmoid volvulus from Africa [6, 7]. Patients in these endemic areas are younger than those in the western countries and predominantly males [17].

Sigmoid colon anatomic dimensions are found at the center of factors predisposing to the development of sigmoid volvulus. Long sigmoid colon and longer than wide meso-sigmoid with narrow mesenteric base attachment are considered important anatomic factors making individuals prone to torsion of their sigmoid colon on its own mesenteric axis [9, 13, 18]. Some studies showed that males unlike females had sigmoid colon anatomic dimensions favoring torsion which could be a reason for the predominance of sigmoid volvulus in males [9, 12, 19].

This study showed that patients for whom an elective sigmoid resection and anastomosis was done for clinically and radiologically confirmed sigmoid volvulus after non-surgical detorsion (group II) had statistically significantly longer sigmoid colon length than those with no past and/or current sigmoid volvulus (group I) and shorter sigmoid colon length than patients for whom emergency abdominal surgery was done for acute sigmoid volvulus and the bowel found viable (group III). Consistent with this study, a previous case-control study by Akinkuotu et al. on live patients found that patients with sigmoid volvulus had longer sigmoid colon length than those with no volvulus [12]. Atamanalp SS. et al. also published a finding that patients with history of volvulus had longer sigmoid colon than those with no history of sigmoid volvulus [13]. These studies, however included emergency sigmoid volvulus cases that acute sigmoid obstruction could contribute to the differences in the anatomic dimensions, an important confounding factor controlled in the current study by using group II and group III as separate comparison groups. Many authors also considered elongated sigmoid colon as an important factor in the development of sigmoid volvulus [9, 20–22]. A long and redundant colon can also predispose individuals to increased bowel complaints like constipation and bloating [23].

Group II patients did have a statistically significantly longer and wider sigmoid mesentery than group I patients. They had averagely shorter and narrower meso-sigmoid than group III patients, but the differences did not amount to statistical significance possibly showing that the effect of acute obstruction might be more marked on sigmoid length and diameter than meso-sigmoid height and width. Generally, this study showed that patients with past and/or current sigmoid volvulus had a longer and wider meso-sigmoid than those with no history of volvulus which is consistent with previous studies [12]. Atamanalp et al also reported a longer sigmoid mesentery in patients with history of sigmoid volvulus, but the width of the mesentery was not significantly different [13].

There was no statistically significant difference in the mesenteric root width among the three groups of this study. In a study on live patients (26 cases with sigmoid volvulus and 12 controls), Akinkuotu et al. [12] reported a finding consistent with this study revealing that patients with sigmoid volvulus had a longer and wider sigmoid mesentery with averagely constant base or root than controls, rather than a longer meso-sigmoid with narrower base as reported in other studies [9, 13, 18].

Meso-sigmoid height to meso-sigmoid maximal width ratio was found to have no significant difference among the study groups. This might be due to the presence of proportional change in meso-sigmoid width in the same direction with meso-sigmoid length in all of the three groups of the study. In line with this finding, Akinkuotu et al. reported that the ratio of the meso-sigmoid maximal width to the meso-sigmoid height was not statistically different between patients with sigmoid volvulus and controls [12], but contrary to this, Antamanalp et al. compared the sigmoid anatomic dimensions of fourteen patients having history of volvulus to those of 433 patients with no history of volvulus and showed that the meso-sigmoid height to meso-sigmoid width ratio was higher in patients with a history of volvulus than those with no history of volvulus [13].

This study also showed that patients with past and/ or current sigmoid volvulus had higher meso-sigmoid height to meso-sigmoid root width and meso-sigmoid maximal width to meso-sigmoid root width ratios than patients having no sigmoid volvulus. The base of the meso-sigmoid of patients with sigmoid volvulus is narrower when seen relative to its height and width. This supported the findings by Atamanalp et al that showed patients with history of sigmoid volvulus had a higher meso-sigmoid height to meso-sigmoid root width ratio [13] and the one reported by Akinkuotu et al that reported patients with sigmoid volvulus had lower meso-sigmoid root width to meso-sigmoid height and meso-sigmoid root width to meso-sigmoid maximal width ratios [12].

The authors want to admit that the study has some limitations. Even though the study showed that there were no differences in their average age and BMI and most came from rural residence, the comparison groups were not matched for factors that might have effects on sigmoid colon anatomic dimensions like differences in ethnicity, dietary and bowel movement habits, and constipation; nor were the effect of these factors assessed. In addition, though senior residents in general surgery after one-day training were the data collectors, interobserver differences in taking the measurements might still present. Future studies should focus on the effect of sigmoid colon anatomic dimensions on patients’ prognoses like post-operative complications and risk of emergency surgery, bowel perforation/necrosis, and recurrences.

## 5. Conclusion

Patients with sigmoid volvulus were found to have statistically significantly longer sigmoid colon, longer and wider meso-sigmoid, and higher meso-sigmoid height to meso-sigmoid root width and meso-sigmoid maximal width to meso-sigmoid root width ratios. No statistically significant difference in sigmoid mesenteric root width was found among the study groups. A long sigmoid colon with long and wide mesentery, but with a constant base is highly likely to predispose individuals to sigmoid volvulus.

## Supporting information

S1 Fig Sigmoid colon and meso-sigmoid splayed-out during measurements. (PDF)

S1 Table Age and sigmoid colon anatomic dimensions of participants based on groups. (PDF)

S2 Table Indications for surgery of group 1 participants. (PDF)
