# Supplementary figures and images for: The role of sigmoid colon anatomic dimensions in the development of sigmoid volvulus, North-Western Ethiopia
Source: PLoS One. 2021 Dec 1;16(12):e0260708. doi: 10.1371/journal.pone.0260708 (PMC8635388; doi:10.1371/journal.pone.0260708)

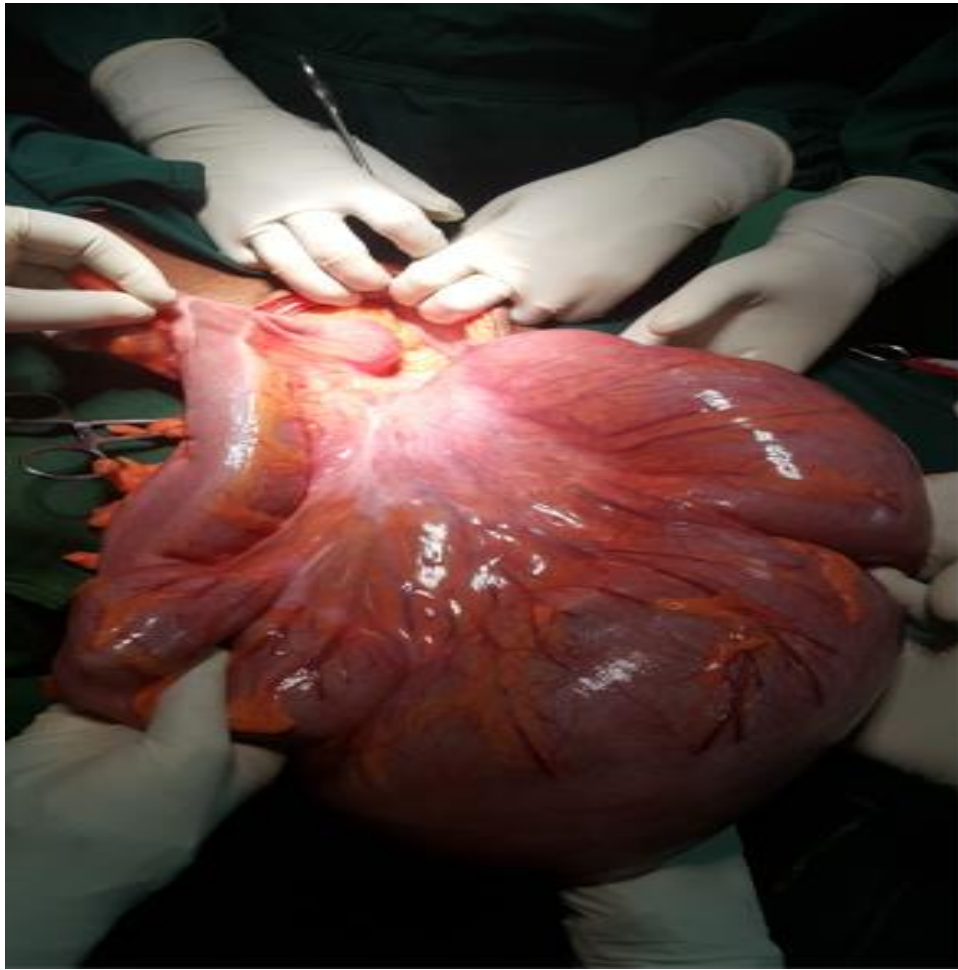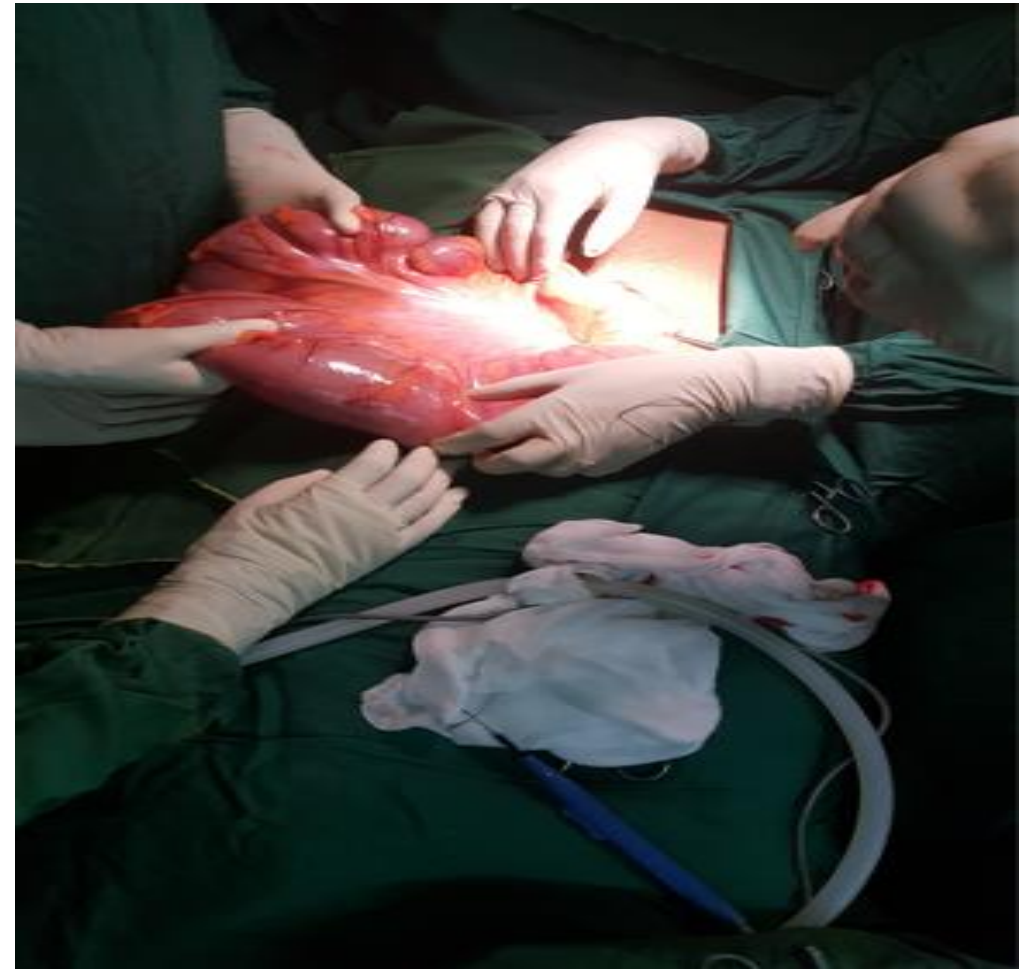

**S1 Fig. Sigmoid colon and meso-sigmoid splayed-out during measurements.**

Supplement: S1 Fig — (PDF) [file pone.0260708.s001.pdf]
