# Supplementary material for: The role of sigmoid colon anatomic dimensions in the development of sigmoid volvulus, North-Western Ethiopia
Source: PLoS One. 2021 Dec 1;16(12):e0260708. doi: 10.1371/journal.pone.0260708 (PMC8635388; doi:10.1371/journal.pone.0260708)
Supplement: S1 Table — (PDF) [file pone.0260708.s002.pdf]

**S1 Table. Age and sigmoid colon anatomic dimensions of participants based on groups**

| Group | Code no. | Age (in year) | Sigmoid anatomic dimensions (in cm) | | | |
|---|---|---|---|---|---|---|
| | | | SCL | MSH | MSMW | MSRW |
| I | 1 | 58.00 | 36.00 | 15.00 | 5.50 | 14.00 |
| | 2 | 65.00 | 35.00 | 12.00 | 8.00 | 12.00 |
| | 3 | 50.00 | 51.00 | 20.00 | 11.00 | 10.00 |
| | 4 | 50.00 | 40.00 | 20.00 | 9.00 | 8.00 |
| | 5 | 60.00 | 50.00 | 21.00 | 11.00 | 6.50 |
| | 6 | 20.00 | 36.50 | 16.00 | 9.00 | 10.00 |
| | 7 | 60.00 | 24.00 | 20.00 | 8.50 | 9.00 |
| | 8 | 64.00 | 34.00 | 16.00 | 5.00 | 10.00 |
| | 9 | 28.00 | 60.00 | 28.00 | 12.00 | 9.50 |
| | 10 | 70.00 | 40.00 | 19.00 | 12.00 | 7.00 |
| | 11 | 38.00 | 45.00 | 19.00 | 13.00 | 10.00 |
| | 12 | 32.00 | 29.00 | 13.00 | 9.50 | 6.00 |
| | 13 | 40.00 | 30.00 | 13.00 | 7.00 | 6.00 |
| | 14 | 20.00 | 37.00 | 18.00 | 11.00 | 7.00 |
| | 15 | 58.00 | 43.00 | 21.00 | 12.00 | 8.00 |
| | 16 | 55.00 | 18.00 | 18.00 | 10.00 | 6.00 |
| | 17 | 21.00 | 33.00 | 14.00 | 13.00 | 9.00 |
| | 18 | 45.00 | 19.00 | 14.00 | 6.00 | 5.00 |
| | 19 | 45.00 | 42.00 | 17.00 | 12.00 | 10.00 |
| | 20 | 52.00 | 38.00 | 17.50 | 11.00 | 7.00 |
| | 21 | 49.00 | 22.00 | 12.00 | 9.00 | 5.00 |
| | 22 | 60.00 | 27.50 | 13.00 | 9.00 | 8.50 |
| II | 1 | 50.00 | 70.00 | 33.00 | 20.00 | 5.00 |
| | 2 | 50.00 | 70.00 | 25.00 | 16.00 | 6.50 |
| | 3 | 70.00 | 75.00 | 30.00 | 18.00 | 8.00 |
| | 4 | 84.00 | 69.50 | 25.50 | 17.00 | 7.00 |
| | 5 | 40.00 | 80.00 | 26.00 | 19.00 | 9.00 |
| | 6 | 60.00 | 69.00 | 22.00 | 12.00 | 8.00 |
| | 7 | 60.00 | 75.00 | 21.00 | 15.00 | 6.00 |
| | 8 | 55.00 | 74.00 | 25.00 | 20.00 | 5.00 |
| | 9 | 58.00 | 65.00 | 22.00 | 14.00 | 8.00 |
| | 10 | 70.00 | 63.00 | 26.00 | 16.00 | 8.00 |
| | 11 | 75.00 | 82.00 | 26.00 | 12.00 | 10.00 |
| | 12 | 35.00 | 67.00 | 23.00 | 10.50 | 6.00 |
| | 13 | 30.00 | 85.00 | 25.00 | 10.00 | 6.00 |
| | 14 | 45.00 | 87.00 | 26.00 | 15.00 | 6.50 |
| | 15 | 75.00 | 55.00 | 31.00 | 17.00 | 7.50 |
| | 16 | 21.00 | 78.00 | 28.00 | 16.00 | 8.00 |
| | 17 | 60.00 | 70.00 | 25.00 | 17.00 | 8.50 |
| | 18 | 80.00 | 70.00 | 29.00 | 9.00 | 5.50 |
| | 19 | 55.00 | 75.00 | 32.00 | 18.00 | 11.00 |

| | | | | | | |
|---|---|---|---|---|---|---|
| | 20 | 55.00 | 56.00 | 27.00 | 13.00 | 9.50 |
| | 21 | 50.00 | 68.00 | 34.00 | 10.00 | 8.00 |
| | 22 | 53.00 | 60.00 | 22.00 | 13.00 | 7.50 |
| III | 1 | 50.00 | 72.00 | 24.00 | 16.00 | 8.00 |
| | 2 | 60.00 | 81.00 | 30.00 | 25.00 | 13.00 |
| | 3 | 65.00 | 88.00 | 32.00 | 19.00 | 11.00 |
| | 4 | 62.00 | 75.00 | 28.00 | 17.00 | 8.00 |
| | 5 | 55.00 | 78.00 | 29.00 | 20.00 | 9.00 |
| | 6 | 33.00 | 69.00 | 23.00 | 17.50 | 6.50 |
| | 7 | 75.00 | 90.00 | 36.00 | 22.00 | 6.50 |
| | 8 | 60.00 | 91.00 | 30.00 | 21.00 | 7.00 |
| | 9 | 30.00 | 93.00 | 33.00 | 16.00 | 10.00 |
| | 10 | 60.00 | 98.00 | 30.00 | 14.00 | 10.00 |
| | 11 | 22.00 | 86.00 | 35.00 | 15.00 | 9.50 |
| | 12 | 40.00 | 78.00 | 28.00 | 18.00 | 6.00 |
| | 13 | 70.00 | 80.00 | 29.00 | 18.00 | 7.00 |
| | 14 | 50.00 | 76.00 | 24.00 | 20.00 | 9.00 |
| | 15 | 46.00 | 60.00 | 28.00 | 11.00 | 7.00 |
| | 16 | 48.00 | 60.00 | 25.00 | 12.00 | 6.00 |
| | 17 | 58.00 | 82.00 | 30.00 | 14.00 | 7.00 |
| | 18 | 55.00 | 75.00 | 33.00 | 18.00 | 12.00 |
| | 19 | 70.00 | 90.00 | 31.00 | 12.00 | 6.00 |
| | 20 | 45.00 | 92.00 | 23.00 | 14.00 | 7.00 |
| | 21 | 25.00 | 80.00 | 28.00 | 13.00 | 7.00 |
| | 22 | 70.00 | 85.00 | 26.00 | 17.00 | 6.00 |
