# Supplementary material for: The role of sigmoid colon anatomic dimensions in the development of sigmoid volvulus, North-Western Ethiopia
Source: PLoS One. 2021 Dec 1;16(12):e0260708. doi: 10.1371/journal.pone.0260708 (PMC8635388; doi:10.1371/journal.pone.0260708)
Supplement: S2 Table — (PDF) [file pone.0260708.s003.pdf]

S2 Table. Indications for surgery of group 1 participants

Code no.	Indications for surgery
1	Gastri ca
2	Gastro-esophageal junction (GEJ) tumor
3	Retro-peritoneal mass
4	Gastric outlet obstruction (GOO)/chronic PUD
5	Gastric outlet obstruction (GOO)/chronic PUD
6	Gastric outlet obstruction (GOO)/chronic PUD
7	Gastric outlet obstruction (GOO)/chronic PUD
8	Gastric outlet obstruction (GOO)/chronic PUD
9	Gastric outlet obstruction (GOO)/chronic PUD
10	Ascending colon ca
11	GEJ tumor
12	Gastric ca
13	Small bowel mesenteric cyst
14	Gastric outlet obstruction (GOO)/chronic PUD
15	Small bowel mesenteric cyst
16	Small bowel mesenteric cyst
17	Negative Laparotomy
18	Right adrenal mass
19	Gastric outlet obstruction (GOO)/chronic PUD
20	Gastric ca
21	Retro-peritoneal mass
22	Gastric ca
